# Usability of Smart Home Thermostat to Evaluate the Impact of Weekdays and Seasons on Sleep Patterns and Indoor Stay: Observational Study

**DOI:** 10.2196/28811

**Published:** 2022-04-01

**Authors:** Niloofar Jalali, Kirti Sundar Sahu, Arlene Oetomo, Plinio Pelegrini Morita

**Affiliations:** 1 School of Public Health and Health Systems University of Waterloo Waterloo, ON Canada; 2 Institute of Health Policy, Management, and Evaluation University of Toronto Toronto, ON Canada; 3 Department of Systems Design Engineering University of Waterloo Waterloo, ON Canada; 4 eHealth Innovation, Techna Institute University Health Network Toronto, ON Canada

**Keywords:** public health, Internet of Things (IoT), big data, sleep monitoring, health monitoring, mobile phone

## Abstract

**Background:**

Sleep behavior and time spent at home are important determinants of human health. Research on sleep patterns has traditionally relied on self-reported data. Not only does this methodology suffer from bias but the population-level data collection is also time-consuming. Advances in smart home technology and the Internet of Things have the potential to overcome these challenges in behavioral monitoring.

**Objective:**

The objective of this study is to demonstrate the use of smart home thermostat data to evaluate household sleep patterns and the time spent at home and how these behaviors are influenced by different weekdays and seasonal variations.

**Methods:**

From the 2018 ecobee *Donate your Data* data set, 481 North American households were selected based on having at least 300 days of data available, equipped with ≥6 sensors, and having a maximum of 4 occupants. Daily sleep cycles were identified based on sensor activation and used to quantify sleep time, wake-up time, sleep duration, and time spent at home. Each household’s record was divided into different subsets based on seasonal, weekday, and seasonal weekday scales.

**Results:**

Our results demonstrate that sleep parameters (sleep time, wake-up time, and sleep duration) were significantly influenced by the weekdays. The sleep time on Fridays and Saturdays is greater than that on Mondays, Wednesdays, and Thursdays (n=450; *P*<.001; odds ratio [OR] 1.8, 95% CI 1.5-3). There is significant sleep duration difference between Fridays and Saturdays and the rest of the week (n=450; *P*<.001; OR 1.8, 95% CI 1.4-2). Consequently, the wake-up time is significantly changing between weekends and weekdays (n=450; *P*<.001; OR 5.6, 95% CI 4.3-6.3). The results also indicate that households spent more time at home on Sundays than on the other weekdays (n=445; *P*<.001; OR 2.06, 95% CI 1.64-2.5). Although no significant association is found between sleep parameters and seasonal variation, the time spent at home in the winter is significantly greater than that in summer (n=455; *P*<.001; OR 1.6, 95% CI 1.3-2.3). These results are in accordance with existing literature.

**Conclusions:**

This is the first study to use smart home thermostat data to monitor sleep parameters and time spent at home and their dependence on weekday, seasonal, and seasonal weekday variations at the population level. These results provide evidence of the potential of using Internet of Things data to help public health officials understand variations in sleep indicators caused by global events (eg, pandemics and climate change).

## Introduction

### Background

Sleep is vital for human health, as it promotes physical and mental well-being at the individual and population levels [1]. Sleep affects brain function and the performance of other systems within the body such as digestive, cardiovascular, and endocrine [2,3]. The lack of proper sleep can cause fatigue, reduced concentration, and depression [4]. In addition, inefficient or disturbed sleep, due to behaviors such as technology use (eg, use of mobile device screens), can also lead to chronic stress and poor mental health [5]. Researchers have found that insufficient sleep is associated with a significant increase in mortality, diabetes, cardiovascular disease, coronary heart disease, and obesity [6]. Today, sleep health is understood not as an isolated portion of the day but as a significant part of a healthy 24-hour cycle [7].

The Public Health Agency of Canada recommends that individuals aged 18 to 64 years get between 7 and 9 hours of sleep per night and those aged ≥65 years between 7 and 8 hours per night [8]. In Canada, at least one in four adults is not getting enough sleep [8]. Even higher levels of sleep deprivation are reported for those aged 35 to 64 years. Similarly, in the United States, 25% of adults self-reported having <7 to 9 hours of sleep [9]. Insufficient sleep duration occurs despite the time spent indoors having increased over the last century [10].

Sleep research has historically relied on single-sleep questionnaires or sleep diaries [11]. However, self-reported sleep data are prone to recall bias and social desirability bias [11]. Sleep patterns have also been inferred by measuring human brain activity, breathing and blood oxygenation levels, muscle and eye movements, and heart rate [12]. These types of studies, although informative, rely on sleep data collected from artificial laboratory environments and do not reflect sleep patterns in a real-world setting, as individuals are often sleeping in a controlled environment with different, albeit fewer, disturbances. There is a need for the modernization of research methodologies to enable the unbiased collection of sleep pattern data in real-world settings.

The development of smartphones and wearable devices has enabled continuous behavioral monitoring [9]. The assessment of sleep behavior can now be performed using wearable devices such as smartwatches or actigraphs [11] or by interpreting the interactions of a user with the device (eg, smartphone screen) [4].

Advances in smart home technology and the Internet of Things (IoT) have the potential to take behavioral monitoring even further [13]. Smart devices collect data objectively; they are unobtrusive and require zero effort from study participants [14]. This technology has the additional advantage that it can assess behavior in individuals with physical or mental impairments who may be unable to interact with smartphones or wearable devices [15]. These advances offer a previously unprecedented opportunity to monitor sleep behaviors in a real-world setting using methods that can reduce participant bias [16]. Previous studies have successfully used smart thermostats to monitor indoor behavior including sleep [17].

### Objectives

The objective of this study is to evaluate the potential use of smart home thermostats to help us understand population-wide sleep patterns, as well as the time the population spends indoors during the year. To assess the impact of different seasonal patterns (eg, days of the week, weekdays vs weekends, and seasons of the year) on sleep health as well as indoor activity, we developed the IoT-based population-level indicators for sleep duration, sleep time, wake-up time, and time spent at home. These indicators are compared across multiple seasonal patterns as weekdays, seasons of the years, and a combined cross-analysis between weekdays and seasons of the year. Ultimately, this study will provide evidence of the potential use of large-scale IoT data to help public health researchers understand the sleep patterns of their population by using the nonobtrusive data collection methods, which will lead to the future use of these data to understand the effects of large-scale global health events (eg, pandemics and climate change).

## Methods

This is an exploratory study using secondary data from IoT devices. In this study, we used smart thermostat data from North America.

### Data

In this study, our team explored the use of the *Donate Your Data (DYD)* data from the ecobee smart home thermostat. The data are composed of the anonymized indoor activity of households captured every 5 minutes through the embedded motion sensors [18]. Approximately 98% of participating households in the DYD program are in North America. Taking the specification of ecobee motion sensors into account, for a family size of up to 4, the distribution of floor area has been identified. On the basis of previous exploratory work, we identified the optimum number of sensors based on the average floor area and minimum distance coverage of the sensors. Our household selection criteria included a minimum of 300 days of data available on the data set, the presence of at least six motion sensors in the home, and a maximum of 4 residents. The data management and analysis have been performed on Microsoft Azure Databricks and the scikit learn library [19] in Python (version 3.6).

### Defining the Sleep Parameters and Time Spent at Home

The original DYD data were reported every 5 minutes. To avoid unnecessary uncertainty, the data were aggregated in 30-minute intervals [18,20,21]. The sum of activation of all sensors, in every 30-minute interval, corresponds to the activity level for that period.

The activation of 1 sensor for 5 minutes corresponds to a score of 1. In a 30-minute interval, the active interval was defined as a score ≥4 (eg, 1 sensor active for 4×5-minute interval [20 minutes], 4 sensors each active for 5 minutes, or any combination of the aforementioned parameters). Intervals with activation sums below this threshold were considered noise. The data were compounded into a binary vector representing a daily record with 48 time slots [18].

This activation pattern was identified to ensure that, while avoiding unnecessary noise, two types of behavior can be picked up by the system: (1) individuals staying in the same room for extended periods, hence activating one sensor sequentially, or (2) individuals moving around the house, hence activating multiple sensors in a shorter time frame.

**Figure 1:**
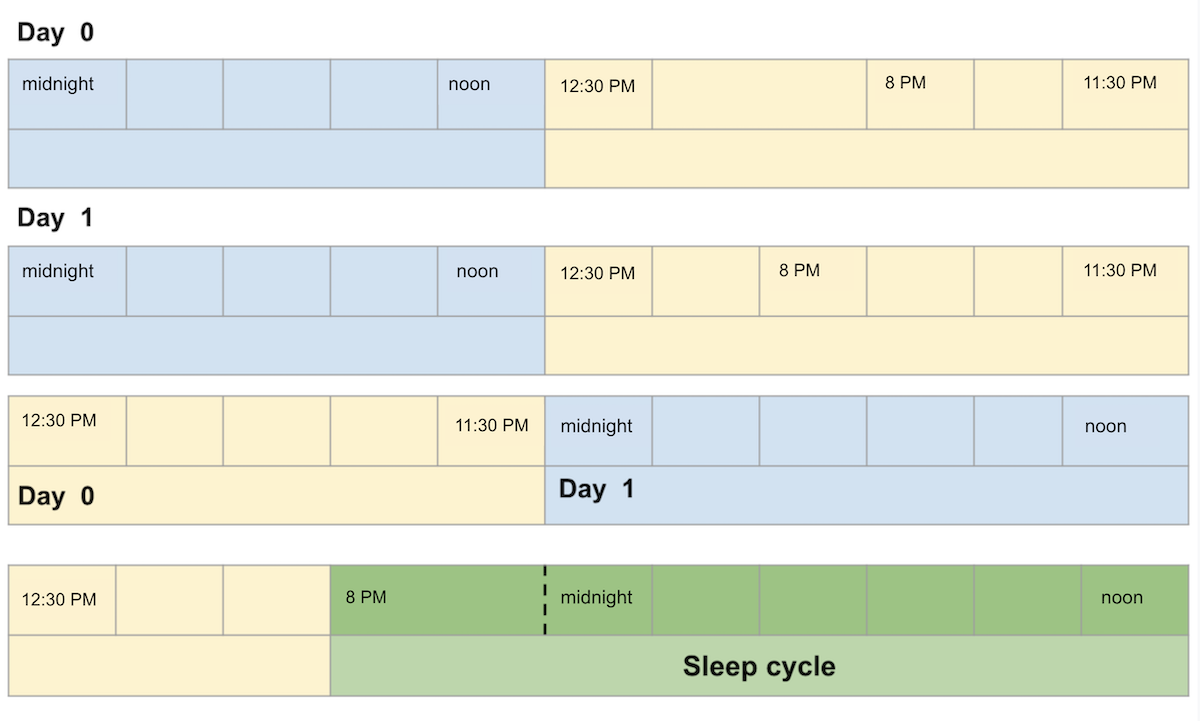
Combining the 2 consecutive dates to create the sleep cycle and identify the sleep time, sleep duration, and wake-up time.

To develop the different sleep indicators discussed above, we divided each day into two parts, namely, (1) midnight until noon and (2) 8 PM to midnight, and disregarded the time interval from noon to 8 PM. In every 2 consecutive days (eg, days 0 and 1), a sleep cycle was defined as the second part of day 0 combined with the first part of day 1 (Figure 1).

To identify the sleep indicators of each household, the following steps were performed:

1. using the Gaussian mixture model [22] to segment the sleep cycle records into different clusters to differentiate the sleep–wake-up behaviors through the selected time scale,
2. identifying the sleep–wake-up patterns in each cluster by averaging the activation of sensors at each time interval (if the average of activation is >0.5, it is assumed that the sensor was active at that time; otherwise, it is assumed as inactive), and
3. specifying the sleep indicators for each sleep–wake-up pattern, using the following assumptions:
  the deactivation (sleep time) occurs before activation (wake-up time),
  the earliest possible deactivation (sleep time) can start at 8 PM, and
  the largest interval between 2 consecutive deactivation and activation times (eg, starts at 8 PM until noon) represents the sleep time, wake-up time, and sleep duration.

Ultimately, the weighted average of each indicator demonstrates the result of sleep parameters for each household at the selected time scale, where the weighted average is defined by the following:



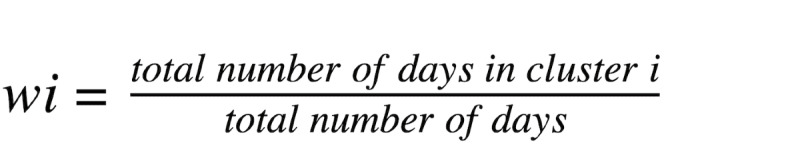



In addition to sleep indicators, we also explored the amount of time spent at home, where the daily cycle records are used to identify the different patterns in each cluster. From each pattern, the amount of time spent at home is defined by the sum of activation of sensors at each interval. The weighted average of each cluster demonstrates the ultimate result.

To explore some of the seasonal effects on sleep and indoor activity patterns, we stratified the data based on different time frames: weekdays and seasons of the year. Next, we compared the different indicators in each of the different time frames (ie, sleep and time spent at home). For each stratified group, the indicators were calculated and the statistical significance between groups was evaluated using analysis of variance (ANOVA) [23]. The statistically significant differences of two indicators (ie, sleep and time at home) were further explored using Tukey post hoc tests [24].

For each of the stratified groups, we present complete descriptive statistics: sample size, mean (SD), SE, and 99% CI of the mean. We assumed that the subsets are independent and distributed normally and the variances are homogeneous [25]. The homogeneity of variance was evaluated using the Levene test [25].

### Seasons of the Year

Data were stratified by seasons of the year, dividing the annual record of each household into 4 seasons [26]. The start and end dates of each season are as follows: winter (January 1 to March 21), spring (March 22 to June 21), summer (June 22 to September 21), and fall (September 22 to December 21).

The distribution of all the subsets as well as the homogeneity of variances was checked using the Levene test [23,27]. The 1-way ANOVA and Tukey post hoc tests were used to evaluate the statistically significant relationship between sleep time, wake-up time, sleep duration, and time spent at home with respect to different seasons.

### Weekdays

A similar approach was used for stratifying the data on different weekdays. The annual records of each household were stratified into 7 subsets, representing data from each weekday. The distribution of all the subsets, as well as the homogeneity of variances, was investigated using the Levene test. The 1-way ANOVA and Tukey post hoc tests were used to evaluate the statistically significant relationship among sleep time, wake-up time, sleep duration, and time spent at home when comparing different weekdays.

### Seasons of the Year and Weekdays

In the combined analysis exploring seasons of the year and weekdays, the annual record of each household was divided into two independent variables: each of 4 seasons and each of 7 days of the week.

The same approach has been replicated for the analysis of different sleep parameters and time spent at home. The 2-way ANOVA [23] and Tukey post hoc test were used to compare the differences of each parameter for different seasons and weekdays simultaneously.

## Results

### Overview

To examine sleep patterns across large populations, we explored the *DYD* ecobee smart home thermostat data set. The DYD program is hosted by ecobee and provides researchers with access to anonymized data from 110,000 households. After we assessed these households for eligibility, a total of 481 households met the inclusion criteria and were included in this study: they had at least 300 days of data available in the DYD data set, at least six sensors, and a maximum of 4 residents. Of the 481 households included in the study, 390 (81.1%) were in the United States, 63 (13.1%) were in Canada, and 28 (5.8%) had undeclared locations. The largest proportion (40/390, 10%) of households in the United States were in the state of California. In Canada, most households were from the province of Ontario (40/63, 65%).

### Effect of Seasons of the Year on Sleep Parameters and Time Spent at Home

To examine the effect of different seasons on sleep parameters, household data were stratified into 4 seasons. For each of the four indicators (sleep time, wake-up time, sleep duration, and time spent at home), the statistical distributions are presented in Table 1.

The sleep duration and time spent at home, is presented as the total number of minutes. While, the sleep time and wake-up time are presented using the standard hh:mm format.

Knowing that the null hypothesis of the Levene test is that the groups we are comparing all have equal population variances, the results would confirm the homogeneity of variances in each of the stratified groups is (Table 2).

We can declare all groups homogeneous with a significance threshold of (*P*<.01). A 1-way ANOVA test was used to compare the seasonal differences for different sleep parameters and time spent at home (Table 3).

Assuming that all other variables are constant, there is a statistically significant difference in time spent at home in different seasons of the year. However, season alone has no statistically significant impact on sleep time, wake-up time, and sleep duration.

The Tukey post hoc test was performed to identify the significant pairs. The difference in means, CIs, and adjusted *P* values per pair are presented in Table 4.

The results indicate a significant difference in the time spent at home between winter and summer (*P*<.001; odds ratio [OR] 1.6, 95% CI 1.3-2.3).

The results of the Tukey post hoc test demonstrate that the time spent at home among these households during the winter is statistically significant from that during the summer. On average, individuals in these households spend, in the summer, 1 extra hour outside when compared with that in the winter. These results demonstrate the potential of this IoT data set to inform public health practice by providing insights on population-level behaviors in different conditions.

**Table 1:** Descriptive statistics for season-based stratified groups for sleep parameters (sleep time, wake-up time, and sleep duration) and time spent at home.^a^

Season		Value, N	Value, mean (SD; SE; 99% CI)
**Sleep time**			
	Spring	461	22:32 (98.5; 4.59; 22:20-22:44)
	Summer	455	22:32 (108.37; 5.08; 22:19-22:45)
	Fall	461	22:25 (89.12; 4.15; 22:14-22:36)
	Winter	466	22:35 (107.11; 4.96; 22:22-22:48)
**Wake-up time**			
	Spring	461	6:22 (93.05; 4.33; 6:11-6:33)
	Summer	455	6:31 (84.75; 3.97; 6:21-6:41)
	Fall	461	6:26 (88.36; 4.12; 6:15-6:37)
	Winter	466	6:26 (102.75; 4.76; 6:14-6:39)
**Sleep duration**			
	Spring	461	466.54 (106.23; 4.95; 453.79-479.28)
	Summer	455	472.54 (109.59; 5.14; 459.31-485.77)
	Fall	461	477.83 (106.2; 4.95; 465.09-490.57)
	Winter	466	465.05 (110.65; 5.13; 451.84-478.25)
**Time spent home**			
	Spring	461	523.13 (227.56; 10.6; 495.83-550.43)
	Summer	455	486.58 (237.4; 11.13; 457.91-515.25)
	Fall	461	529.39 (226.42; 10.55; 502.22-556.55)
	Winter	466	546.43 (235.53; 10.91; 518.32-574.53)

^a^The mean of sleep time and wake-up time, along with their CIs, is presented using a standard hh:mm format. The SD of all 4 indicators, as well as the sleep duration and time spent at home, is presented as the total number of minutes.

**Table 2:** Evaluating the homogeneity of variances in different seasonal groups using the Levene test.

Indicator	*P* value
Wake-up time	.47
Sleep time	.12
Sleep duration	.84
Time spent home	.66

**Table 3:** Analysis of variance test to explore the effect of seasons of the year on sleep time, sleep duration, wake-up time, and time spent at home.

		Sum of squares	*df*	*F* test	*P* value
**Sleep time**					
	C(season)	26,092.90	3	0.85	.47
	Residual	18,783,017.85	1839		
**Wake-up time**					
	C(season)	20,683.21	3	0.81	.49
	Residual	15,744,147.50	1839		
**Sleep duration**					
	C(season)	13.26	3	1.36	.25
	Residual	5979.04	1839		
**Time spent home**					
	C(season)	874,376.67	3	5.43	<.001
	Residual	98,783,401.15	1839		

**Table 4:** The Tukey post hoc test comparing the statistical significance of the difference between each pair of seasons.

	Group 1	Group 2	Difference (SE)	95% CI of the mean	*q* value	*P* value
**Sleep time**						
	Winter	Spring	3.17 (−4.29)	−13.9 to 20.24	0.68	.90
	Winter	Summer	2.89 (2.96)	−14.24 to20.01	0.61	.90
	Winter	Fall	10.21 (−13.54)	−6.86 to 27.28	2.17	.42
	Summer	Spring	0.28 (−7.25)	−16.89 to 17.46	0.06	.90
	Fall	Spring	7.04 (9.25)	−10.08 to 24.15	1.49	.69
	Fall	Summer	7.32 (16.49)	−9.85 to 24.49	1.55	.67
**Wake-up time**						
	Winter	Spring	4.71 (−10.26)	−10.92 to 20.34	1.10	.85
	Winter	Summer	4.78 (−17.33)	−10.9 to 20.46	1.11	.85
	Winter	Fall	0.47 (−12.22)	−15.16 to 16.10	0.11	.90
	Summer	Spring	9.49 (7.07)	−6.24 to 25.21	2.19	.41
	Fall	Spring	4.24 (1.96)	−11.43 to 19.91	0.98	.89
	Fall	Summer	5.25 (−5.11)	−10.48 to 20.97	1.21	.80
**Sleep duration**						
	Winter	Spring	1.49 (−4.42)	−16.79 to 19.76	0.30	.90
	Winter	Summer	7.49 (−1.06)	−10.84 to 25.83	1.49	.70
	Winter	Fall	12.78 (−4.45)	−5.50 to 31.05	2.54	.27
	Summer	Spring	6.00 (−3.36)	−12.38 to 24.39	1.19	.81
	Fall	Spring	11.29 (0.03)	−7.03 to 29.61	2.24	.39
	Fall	Summer	5.29 (3.39)	−13.1 to 23.67	1.05	.87
**Time spent home**						
	Winter	Spring	23.3 (−7.65)	−15.85 to 62.45	2.16	.42
	Winter	Summer	59.85 (−1.90)	20.57 to 99.13	5.54	<.001
	Winter	Fall	17.04 (−10.18)	−22.11 to 56.19	1.58	.66
	Summer	Spring	36.55 (−5.75)	−2.83 to 75.94	3.38	.08
	Fall	Spring	6.26 (2.54)	−33.00 to 45.51	0.58	.90
	Fall	Summer	42.81 (8.29)	3.43 to 82.19	3.95	.03

### Effect of Different Weekdays Sleep Parameters and Time Spent at Home

The ability to identify sleep indicators and time spent at home on different weekdays, using an IoT data set, is further evidence of the potential use of these data to understand and monitor the behaviors of a population [28]. To examine the influence of different weekdays on the 4 indicators, household data were divided between 7 weekdays.

For each indicator, the descriptive statistics for each weekday as well as the homogeneity of variances within different weekdays are presented in Tables 5 and 6, respectively.

**Figure 2:**
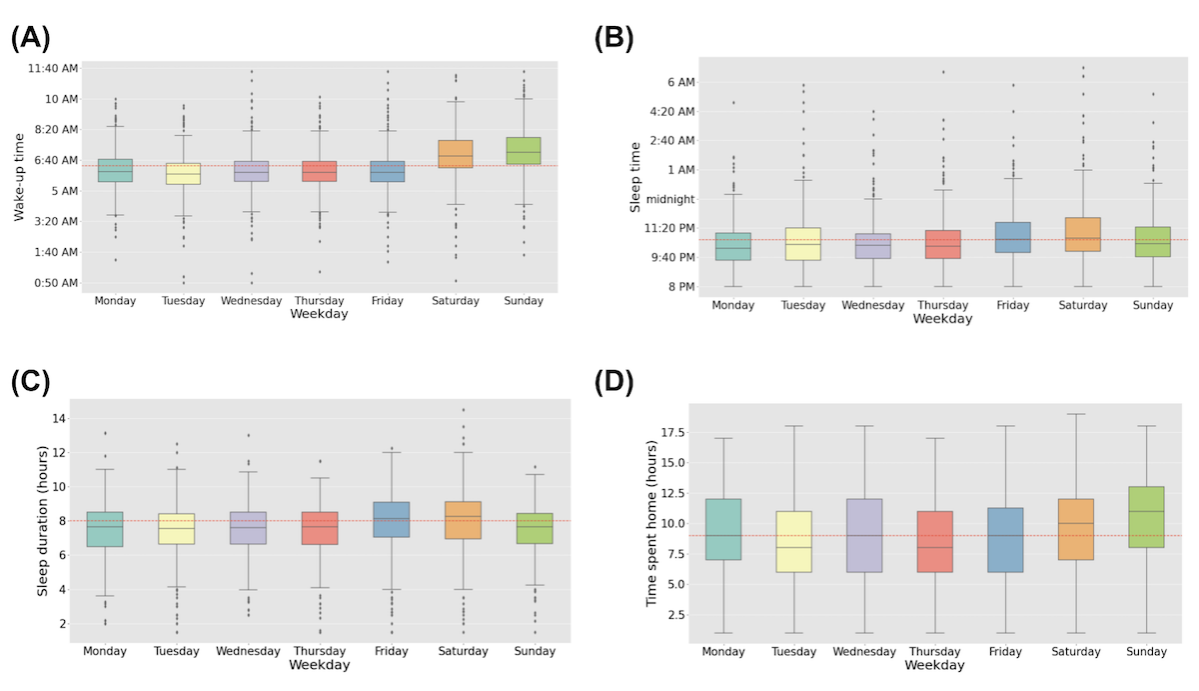
The box plot of wake-up time (A), sleep time (B), sleep duration (C), and time spent at home (D) for weekdays. The dashed line represents the total average.

We found no significant difference of variances among the 7 weekdays for wake-up time, sleep time, and time spent at home. However, the sleep duration is not fulfilling the variance homogeneity assumption, and the results need to be generalized with precaution. As explained in Figure 1, a typical sleep cycle is spread across 2 days, beginning and ending on different dates. Therefore, the sleep time and sleep duration occur on one day and wake-up time on the next day (Figure 1), which is likely one of the reasons for the nonhomogeneous distribution of variances. The sleep time, wake-up time, sleep duration, and time spent at home on weekdays are illustrated in Figure 2.

The average sleep time for the entire sample was 10:40 PM (Figure 2A). Most households had a sleep time earlier than 10:40 PM on Mondays, Tuesdays, Wednesdays, Thursdays, and Sundays. However, 50.1% (232/463) and 52.8% (245/464) of the households had sleep time of equal to or later than 10:40 PM on Fridays and Saturdays, respectively.

The average wake-up time of all the households in the entire sample was 6:20 AM (Figure 2B). Most households have a wake-up time earlier than 6:20 AM during weekdays, and the average wake-up time on weekends was greater than 6:20 AM (Figure 2B).

The average sleep duration for the entire sample was 8 hours (Figure 2C). The average sleep duration of the households was 7½ hours on Mondays, Tuesdays, Wednesdays, Thursdays, and Sundays. The sleep duration was longer than 8 hours on Fridays and Saturdays.

The average time spent at home for the entire sample was 9 hours (Figure 2D). The time spent at home on weekends was >9 hours.

The 1-way ANOVA showed statistically significant differences in sleep indicators and time spent at home for the stratified weekdays, assuming that all other variables were constant (Table 7).

The Tukey post hoc test comparing sleep time, sleep duration, wake-up time, and time spent at home for different weekday pairs are presented in Table 8. The results of the overall comparison and the significant pairs are presented in Multimedia Appendix 1 and Table 8, respectively. Owing to a large number of comparisons, we only present the statistically significant results in Table 8. Out of all the 21 possible pair combinations for each indicator, we had 8 (38%) statistically significant pairs when comparing sleep time, 10 (47%) when comparing wake-up time, 10 (47%) when comparing sleep duration, and 6 (29%) when comparing time spent at home.

The Tukey post hoc test provides evidence that the sleep time on Fridays and Saturdays was statistically different from that on Mondays, Wednesdays, and Thursdays (OR 1.8, 95% CI 1.5-3; *P*<.001). The most significant difference in sleep time was between Mondays and Saturdays, with an average of 40 minutes earlier on Mondays than on Saturdays (Table 8).

There was a statistically significant difference in wake-up time on Saturdays and Sundays compared with that on the remaining weekdays (OR 5.6, 95% CI 4.3-6.3; *P*<.001). The maximum wake-up time difference was between Tuesdays and Sundays, with households waking an average of 76 minutes later on Sundays (Table 8).

The sleep duration on Fridays and Saturdays was statistically significant from the other days of the week (OR 1.8, 95% CI 1.4-2; *P*<.001), with households sleeping longer on Fridays and Saturdays than on the other days of the week. The highest sleep duration difference was between Tuesdays and Saturdays, with an extra 36 minutes of sleep on Saturdays (Table 8).

There was a statistically significant difference in the time spent at home on Sundays with respect to other days of the week (OR 2.06, 95% CI 1.64-2.5; *P*<.001). The highest difference in time spent at home is between Thursdays and Sundays, with an average of 96 minutes more time spent at home on Sundays (Table 8).

These results indicate that the data collected by IoT smart home sensors can provide evidence of expected differences between sleep time, wake-up time, sleep duration, and time spent at home. These results provide further evidence for using these data to monitor population-level changes caused by global events.

**Table 5:** Descriptive statistics of stratified weekday group for sleep indicators and time spent at home.^a^

			Value, N		Value, mean (SD; SE; 99% CI)
**Sleep time (PM)**					
	Monday	463		10:26 (81.3; 3.78; 10:16-10:35)	
	Tuesday	459		10:35 (94.53; 4.41; 10:23-10:46)	
	Wednesday	458		10:31 (86.22; 4.03; 10:20-10:41)	
	Thursday	460		10:33 (99.67; 4.65; 10:21-10:44)	
	Friday	463		10:54 (89.2; 4.15; 10:43-11:04)	
	Saturday	464		11:03 (104.04; 4.84; 10:51-11:16)	
	Sunday	466		10:36 (85.27; 3.95; 10:26-10:47)	
**Wake-up time (AM)**					
	Monday	463		6:06 (70.6; 3.28; 5:57-6:14)	
	Tuesday	459		5:54 (74.22; 3.46; 5:45-6:02)	
	Wednesday	458		6:03 (73.7; 3.44; 5:55-6:12)	
	Thursday	460		6:04 (84.86; 3.96; 5:54-6:14)	
	Friday	463		6:05 (72.76; 3.38; 5:57-6:14)	
	Saturday	464		7:00 (101.23; 4.71; 6:48-7:12)	
	Sunday	466		7:08 (79.51; 3.68; 6:58-7:17)	
**Sleep duration**					
	Monday	463		449.1 (95.83; 4.45; 437.63-460.57)	
	Tuesday	459		447.76 (97.47; 4.55; 436.04-459.48)	
	Wednesday	458		449.88 (92.13; 4.3; 438.79-460.97)	
	Thursday	460		449.73 (96.21; 4.49; 438.17-461.28)	
	Friday	463		477.99 (110.99; 5.16; 464.7-491.27)	
	Saturday	464		483.25 (111.16; 5.17; 469.92-496.57)	
	Sunday	466		450.18 (89.15; 4.13; 439.54-460.82)	
**Time spent home**					
	Monday	463		516.47 (216.85; 10.08; 490.51-542.43)	
	Tuesday	459		486.14 (215.28; 10.05; 460.26-512.02)	
	Wednesday	458		503.46 (219.69; 10.27; 477.02-529.91)	
	Thursday	460		482.41 (213.16; 9.94; 456.81-508.01)	
	Friday	463		498.95 (223.06; 10.37; 472.25-525.65)	
	Saturday	462		533.51 (215.45; 10.02; 507.69-559.33)	
	Sunday	466		576.27 (220.66; 10.22; 549.94-602.6)	

^a^The mean of sleep time and wake-up time, along with their CIs, is presented using a standard hh:mm format. The SD of all 4 indicators, as well as the sleep duration and time spent at home, is presented as the total number of minutes.

**Table 6:** Evaluating the homogeneity of variances in different weekday groups using the Levene test.

Indicator	*P* value
Wake-up time	.02
Sleep time	.02
Sleep duration	<.001
Time spent home	.86

**Table 7:** The analysis of variance test compares weekdays’ impact on sleep time, sleep duration, wake-up time, and time spent at home.

		Sum of squares	*df*	*F* test	*P* value
**Sleep time**					
	C(wday)^a^	524,447.23	6	10.39	<.001
	Residual	27,150,245.54	3226		
**Wake-up time**					
	C(wday)	2,591,512.52	6	67.05	<.001
	Residual	20,782,158.97	3226		
**Sleep duration**					
	C(wday)	180.43	6	10.97	<.001
	Residual	8843.10	3226		
**Time spent home**					
	C(wday)	2,970,000	6	10.39	<.001
	Residual	15,300,000,000	3224		

^a^wday: weekday.

**Table 8:** The significant results of the Tukey test for sleep time, wake-up time, sleep duration, and time spent home were stratified by different weekdays.

	Group 1	Group 2	Difference (SE)	95% CI of the mean	*q* value	*P* value
**Sleep time**						
	Monday	Friday	29.69 (9.93)	10.34-45.93	6.60	.001
	Monday	Saturday	40.35 (25.11)	20.13-55.74	8.89	.001
	Tuesday	Saturday	28.45 (9.15)	11.10-46.78	6.77	.001
	Wednesday	Friday	23.23 (2.74)	5.15-40.83	5.38	.003
	Wednesday	Saturday	33.90 (17.91)	14.94-50.64	7.66	.001
	Thursday	Friday	21.93 (−9.80)	3.34-38.98	4.95	.007
	Thursday	Saturday	32.60 (5.38)	13.13-48.80	7.24	.001
	Saturday	Sunday	−25.67 (−17.14)	9.36-44.91	6.37	.001
**Wake-up time**						
	Monday	Saturday	51.59 (23.42)	38.89-70.00	14.61	.001
	Monday	Sunday	61.23 (8.25)	46.73-77.77	16.73	.001
	Tuesday	Saturday	66.01 (20.75)	50.9-82.08	17.80	.001
	Tuesday	Sunday	75.65 (5.59)	58.74-89.84	19.93	.001
	Wednesday	Saturday	55.36 (20.83)	40.98-72.17	15.13	.001
	Wednesday	Sunday	65.00 (5.66)	48.81-79.93	17.26	.001
	Thursday	Saturday	54.63 (8.48)	40.38-71.53	14.99	.001
	Thursday	Sunday	64.27 (−6.69)	48.21-79.3	17.11	.001
	Friday	Saturday	53.40 (20.32)	39.13-70.23	14.67	.001
	Friday	Sunday	63.04 (5.15)	46.96-78.00	16.80	.001
**Sleep duration**						
	Monday	Friday	28.32 (16.06)	9.63-48.15	6.26	.001
	Monday	Saturday	32.07 (17.06)	14.88-53.42	7.39	.001
	Tuesday	Friday	32.67 (14.72)	10.93-49.53	6.54	.001
	Tuesday	Saturday	36.42 (15.73)	16.18-54.8	7.67	.001
	Wednesday	Friday	29.19 (19.87)	8.79-47.42	6.07	.001
	Wednesday	Saturday	32.94 (20.88)	14.04-52.68	7.20	.001
	Thursday	Friday	28.42 (16.14)	8.97-47.55	6.11	.001
	Thursday	Saturday	32.17 (17.14)	14.22-52.82	7.25	.001
	Friday	Sunday	−29.79 (−21.29)	8.58-47.04	6.03	.001
	Saturday	Sunday	−33.54 (−22.29)	13.83-52.30	7.17	.001
**Time spent home**						
	Monday	Sunday	57.96 (1.06)	17.64-101.95	5.92	.001
	Tuesday	Sunday	89.32 (1.85)	47.88-132.38	8.90	.001
	Wednesday	Sunday	73.31 (−2.23)	30.53-115.08	7.19	.001
	Thursday	Saturday	53.82 (0.72)	8.79-93.42	5.04	.004
	Thursday	Sunday	95.69 (5.39)	51.63-136.08	9.27	.001
	Friday	Sunday	79.39 (−5.58)	35.16-119.47	7.65	001

### The Effect Combined of Seasons and Weekdays on Sleep Indicators and Time Spent at Home

To further investigate the ability of the smart thermostat IoT data to differentiate patterns in the data and days with unique behavioral patterns, we investigated the combined effect of weekdays and seasons of the year. This proposed analysis will focus on comparing the 4 indicators on different seasons of the year but blocking out analysis by weekday. We first divided the data into 7 weekdays and then, within each weekday, into 4 seasons. Descriptive statistics of the season-weekday groups, as well as the validated results of homogeneity of variances among all subsets for wake-up time, sleep duration, and time spent at home have been provided in Multimedia Appendix 1.

We found no significant difference of variance among subsets, except for the Saturday sleep time, which requires caution when generalizing the results.

The 2-way ANOVA [29] demonstrates the statistically significant differences in sleep indicators and time spent at home with respect to the variation of seasons and weekdays (Table 9).

**Figure 3:**
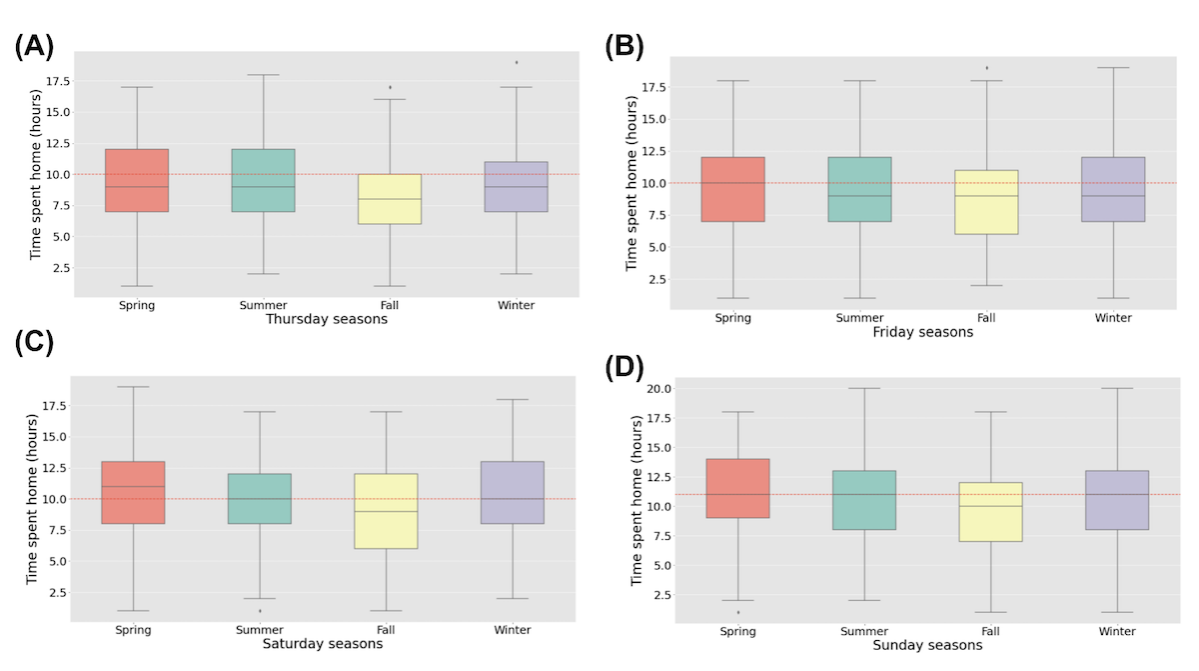
The box plot of time spent at home with respect to different seasons: Thursday seasons (A); Friday seasons (B); Saturday seasons (C); and Sunday seasons (D). The dashed line represents the total average.

The greatest impact of seasonality on weekday-specific variations was seen with respect to time spent at home. Figure 3 illustrates the differences through box plots for Thursdays (Figure 3A), Fridays (Figure 3B), Saturdays (Figure 3C), and Sundays (Figure 3D) with respect to the different seasons. Through ANOVA, followed by the Tukey post hoc test, there was a statistically significant difference in time spent at home on Thursdays in the summer in contrast with other seasons. The average time households spend at home on Thursdays is 8½ hours. The time spent at home during the summer was significantly less than that in all other seasons (Figure 3A–D). The time spent at home in the summer on Fridays (Figure 3B), Saturdays (Figure 3C), and Sundays (Figure 3D) is significantly less than in the winter. The time spent at home on Saturdays in the summer is statistically different from that in winter and fall. Households spend less time at home on Saturdays during the summer than during the fall (Figure 3C).

The results demonstrate that the sleep indicators and the time spent at home are significantly associated with variation of seasons of the year and weekdays. To identify the season’s impact on each weekday and compare the different parameters of sleep and time spent at home, we used the Tukey post hoc test to compare the variation of seasons with respect to the specific weekday. The overall comparison and the significant pair results are presented in the Multimedia Appendix 1 and Table 10, respectively.

Different seasons did not have a statistically significant effect on sleep time and sleep duration. There was a statistically significant difference between the wake-up time on Fridays between summer and winter, with an average of 27 (SD 5) minutes (Table 10).

**Table 9:** The 2-way analysis of variance test to compare season and weekdays’ impact on sleep time, sleep duration, wake-up time, and time spent at home.

		Sum of squares	*df*	*F* test	*P* value
**Sleep time**					
	C(wday)^a^	1,639,363	6	32.18	<.001
	C(season)	136,515.4	3	5.36	.001
	Residual	112,487,100	13,247.0		
**Wake-up time**					
	C(wday)	5,786,750.05	6	134.32	<.001
	C(season)	379,805.86	3	17.63	<.001
	Residual	95,119,438.37	13,247.0		
**Sleep duration**					
	C(wday)	394.48	6	22.47	<.001
	C(season)	135.76	3	15.47	<.001
	Residual	38.752.79	13,247.0		
**Time spent home**					
	C(wday)	9,840,000	6	38.08	<.001
	C(season)	4,550,000	3	35.19	<.001
	Residual	571,000,000	13,247.0		

^a^wday: weekday.

**Table 10:** The significant results of the Tukey test for sleep time, wake-up time, sleep duration, and time spent at home for each weekday but different seasons.

Group 1		Group 2	Difference (SE)	95% CI of the mean	*q* value	*P* value
**Wake-up time**						
	(Winter, Friday)	(Summer, Friday)	27.06 (4.93)	3.48-44.42	6.15	<.001
**Time spent home**						
	(Winter, Thursday)	(Summer, Thursday)	−81.23 (−5.77)	29.61-130.07	8.35	<.001
	(Winter, Friday)	(Summer, Friday)	−60.80 (−3.02)	8.06-108.36	6.10	.01
	(Winter, Saturday)	(Summer, Saturday)	−81.37 (−7.01)	32.01-132.58	8.60	<.001
	(Winter, Sunday)	(Summer, Sunday)	−69.50 (−5.42)	21.78-122.24	7.53	<.001
	(Spring, Thursday)	(Summer, Thursday)	−74.08 (−4.07)	24.00-124.36	7.77	<.001
	(Summer, Thursday)	(Fall, Thursday)	58.16 (−0.72)	10.91-111.00	6.40	<.001
	(Summer, Saturday)	(Fall, Saturday)	63.58 (−11.09)	11.55-111.90	6.46	<.001

## Discussion

### Principal Findings

#### Overview

The advent of smart home technology has provided a previously unprecedented opportunity to collect population-wide, reliable, objective, nonintrusive data on human behavioral patterns. In this study, we evaluated the potential use of ecobee smart home thermostat data as a potential data source for informing public health practice.

#### Validation of IoT Smart Home Data as Indicators of Healthy Behavior

The initial step in the validation of a new data source as an indicator for public health monitoring is to demonstrate the discriminability of the data in the data set [30]. As new indicators are developed, researchers must demonstrate that variations in independent variables (ie, days of the week and seasons) will result in consistent and expected changes in the dependent variables (ie, sleep indicators and time at home) [31,32]. In this study, we successfully demonstrated the variations in our public health indicators of sleep and time at home (Table 8) caused by changes in the dependent variables. In the next few sections, we provide further discussion of the potential benefits of these indicators for public health practice and research.

#### Sleep Parameters and Their Impact on Public Health

Our data showed that the seasons of the year had no impact on sleep time, wake-up time, and sleep duration, assuming that all other variables are constant. Our results are in agreement with previous sleep studies conducted in contemporary Western societies [8,33-36] and indicate that the use of smart home thermostat data is a valid method to examine household sleep patterns. In contrast, a 2018 study conducted in Japan showed that seasons significantly affect the sleep parameters of the adult population [37]. These differences may reflect geo-climatic and sociocultural differences and their potential effects on sleep parameters.

Consistent with other reports [38], we found a strong influence of weekdays on sleep time, wake-up time, and sleep duration (Table 8). The sleep time and sleep duration on Fridays and Saturdays were significantly greater than on the other days of the week (Table 8), which is consistent with other studies in this space [38,39]. There is a statistically significant difference in wake-up time between weekends and weekdays, as also demonstrated by Zhang et al [40]. Understanding influences on sleep patterns is an important health determinant, as short sleep duration on weekdays (weekday sleep debt) is a risk factor for chronic diseases and can lead to early mortality [38].

#### Time Spent Indoors and Its Impact on Public Health

Using the ecobee thermostat data, we also demonstrated that residents spend on average 9 hours per day in their dwellings. Time spent at home varied significantly with respect to different days of the week and season. Previous studies on the impact of different seasons and time spent at home identify that time at home outdoors can vary according to factors such as the seasons, occupation, and age [41,42].

Our results demonstrate a statistically significant difference in the time spent at home between summer and winter, assuming that all other variables are constant (Table 4). In winter, the average daily time spent at home is 1 hour greater than that during the summer (Table 1). Previous studies are consistent with our results, showing a significant difference between physical activity and sedentary behavior between the winter and summer seasons, increasing time indoors during the winter, also found in the literature [33,43-45].

The time spent indoors varies from <8 hours to >10 hours for different days of the week (from a minimum average of 482.41 minutes on Thursdays to a maximum average of 576.27 minutes on Sundays) (Table 5), signifying a strong influence of weekdays and weekend variation on indoor behavior. Significantly more time was spent at home on Sundays than on other days of the week. These effects are consistent with the literature, as demonstrated by Bittman [46].

The duration of staying at home for each individual is linked to physical and mental health [47-49]. More time spent inside the house causes detachment from the natural world, reduced sunlight exposure, lower probability of physical activity, sedentary behavior, exposure to air pollutants, and reduced social interaction [50-52]. Physical activity and sedentary behavior have a strong influence on the risk factors of chronic diseases [53].

#### Seasonal Weekdays Scale

This study is the first to explore the simultaneous effect of seasons and weekdays on sleep parameters and time spent indoors. Our findings show a significant effect on wake-up time and time spent at home, but not on sleep time and sleep duration (Table 10). We identified that the wake-up time on Fridays in the summer is significantly greater than in the winter (Table 10). The average time spent at home in the summer (Thursdays, Fridays, Saturdays, and Sundays) is significantly less than that in the winter, which is consistent with findings from other researchers such as Plasqui and Westerterp [54], Matz et al [55], and Farrow et al. [56]. Residents spend less time at home on Saturdays during the summer than during the fall.

#### Strengths of IoT-Based Public Health Monitoring

Previous studies used self-reported surveys [57], sleep diaries [58], accelerometers [59], wearables [60], and social media [61] to calculate different sleep parameters at the individual and population levels. This study is unique in its use of smart thermostats and remote motion sensors as data sources. The sleep results presented here are consistent with those published using other data sources, highlighting the validity of our methods. These data sets and analysis techniques can monitor behavioral health risk indicators at the population level for different geographic and geopolitical locations.

The use of big data from IoT devices has several advantages over traditional data sources [62]. This analysis used 1 year of data that can be extended to include additional years for the same households, enabling identification of longitudinal, long-term patterns. The total data set includes data from >100,000 households, with >5 years of data. Another strength of this data set is the granularity of the data, which are reported every 5 minutes. The use of zero-effort technology and data integration from several sensors will further enhance public health syndromic surveillance [63], with near–real-time monitoring potentially providing evidence of the immediate effect of public health policies at the population level. The COVID-19 pandemic has provided strong evidence of how our monitoring systems in public health need to be optimized and that the gap between data collection and data use must be reduced [64].

### Conclusions

This study is the first of its kind, leveraging smart home thermostat data to monitor sleep indicators and time spent at home on different temporal scales (weekdays, seasonal, and seasonal weekdays) at the population level. This approach not only uses nonadhesive and zero-effort technology to collect the data but can also improve the decision-making of public health officials through large-scale, near–real-time IoT data. Our results demonstrate the variations in sleep indicators and time spent at home for different days of the week and different seasons of the year, which provides evidence of the discriminability of the data and will potentially lead to wider-scale use of IoT data in public health, which has the potential to monitor the effects of global events such as climate change and pandemics.

### Limitations

Some of the limitations of this study include the absence of household socioeconomic and demographic data. The DYD data set is collected and compiled by ecobee, which unfortunately prevents us from collecting important additional data from our participants. As a result, no stratified analysis is possible for age, sex, race, and health indicators. In addition, the self-reported number of occupants and the size of the house may be inaccurate. As this study uses data at the household level, an individual-level analysis is not possible. Similarly, the data provided by ecobee only include information from indoor movement using sensors, limiting the analysis to indoor behaviors. Integration of these data with other IoT devices such as smartwatches, cell phones, and fitness trackers would provide more comprehensive insights into population-level behaviors.

## Appendix

Multimedia Appendix 1 The Tukey post hoc test compare sleep parameters and time spent at home with respect to different weekday pairs (Table S1); descriptive analysis of seasonal weekdays subgroups for sleep parameters and time spent at home (Table S2); evaluating the homogeneity of variances in different weekdays for all season groups using the Levene test (Table S3).

## Abbreviations

ANOVA: analysis of variance
DYD: Donate Your Data
IoT: Internet of Things
OR: odds ratio
