# Supplementary material for: Usability of Smart Home Thermostat to Evaluate the Impact of Weekdays and Seasons on Sleep Patterns and Indoor Stay: Observational Study
Source: JMIR Mhealth Uhealth. 2022 Apr 1;10(4):e28811. doi: 10.2196/28811 (PMC9015749; doi:10.2196/28811)
Supplement: Multimedia Appendix 1 [file mhealth_v10i4e28811_app1.docx]

**Multimedia Appendix**

Table S1: The Tukey post hoc test compare sleep parameters and time spent at home with respect to different weekday pairs

| group1 | group2 | Diff | Std_Diff | Lower | Upper | q-value | p-value |
| --- | --- | --- | --- | --- | --- | --- | --- |
| **Sleep time** | | | | | | | |
| Mon | Tue | 11.91 | 15.96 | -6.29 | 30.1 | 2.73 | 0.46 |
| Mon | Wed | 6.46 | 7.19 | -11.73 | 24.65 | 1.48 | 0.9 |
| Mon | Thu | 7.76 | 19.73 | -10.41 | 25.93 | 1.78 | 0.86 |
| Mon | Fri | 29.69 | 9.93 | 11.55 | 47.83 | 6.83 | <0.001 |
| Mon | Sat | 40.34 | 25.11 | 22.18 | 58.5 | 9.27 | <0.001 |
| Mon | Sun | 14.68 | 7.97 | -3.44 | 32.8 | 3.38 | 0.2 |
| Tue | Wed | -5.45 | -8.76 | -12.79 | 23.69 | 1.25 | 0.9 |
| Tue | Thu | -4.15 | 3.77 | -14.07 | 22.37 | 0.95 | 0.9 |
| Tue | Fri | 17.79 | -6.03 | -0.4 | 35.98 | 4.08 | 0.06 |
| Tue | Sat | 28.44 | 9.15 | 10.23 | 46.65 | 6.52 | <0.001 |
| Tue | Sun | 2.78 | -7.99 | -15.39 | 20.95 | 0.64 | 0.9 |
| Wed | Thu | 1.3 | 12.54 | -16.92 | 19.52 | 0.3 | 0.9 |
| Wed | Fri | 23.23 | 2.74 | 5.04 | 41.42 | 5.33 | <0.001 |
| Wed | Sat | 33.89 | 17.91 | 15.67 | 52.1 | 7.76 | <0.001 |
| Wed | Sun | 8.22 | 0.78 | -9.95 | 26.4 | 1.89 | 0.81 |
| Thu | Fri | 21.93 | -9.8 | 3.76 | 40.1 | 5.04 | 0.01 |
| Thu | Sat | 32.59 | 5.38 | 14.39 | 50.78 | 7.47 | <0.001 |
| Thu | Sun | 6.92 | -11.76 | -11.23 | 25.07 | 1.59 | 0.9 |
| Fri | Sat | 10.65 | 15.18 | -7.51 | 28.81 | 2.45 | 0.58 |
| Fri | Sun | -15.01 | -1.96 | -3.11 | 33.13 | 3.46 | 0.18 |
| Sat | Sun | -25.66 | -17.14 | 7.52 | 43.8 | 5.9 | <0.001 |
| **Wakeup time** | | | | | | | |
| Mon | Tue | -14.42 | 2.66 | -1.09 | 29.94 | 3.88 | 0.09 |
| Mon | Wed | -3.77 | 2.59 | -11.75 | 19.28 | 1.01 | 0.9 |
| Mon | Thu | -3.04 | 14.94 | -12.46 | 18.54 | 0.82 | 0.9 |
| Mon | Fri | -1.81 | 3.1 | -13.66 | 17.28 | 0.49 | 0.9 |
| Mon | Sat | 51.59 | 23.42 | 35.45 | 66.43 | 13.72 | <0.001 |
| Mon | Sun | 61.23 | 8.25 | 45.78 | 76.69 | 16.53 | <0.001 |
| Tue | Wed | 10.65 | -0.08 | -4.91 | 26.21 | 2.86 | 0.4 |
| Tue | Thu | 11.38 | 12.28 | -4.16 | 26.92 | 3.06 | 0.32 |
| Tue | Fri | 12.61 | 0.44 | -2.9 | 28.13 | 3.39 | 0.2 |
| Tue | Sat | 66.01 | 20.75 | 49.83 | 80.9 | 17.56 | <0.001 |
| Tue | Sun | 75.65 | 5.59 | 60.16 | 91.15 | 20.37 | <0.001 |
| Wed | Thu | 0.73 | 12.35 | -14.81 | 16.27 | 0.2 | 0.9 |
| Wed | Fri | 1.96 | 0.51 | -13.55 | 17.48 | 0.53 | 0.9 |
| Wed | Sat | 55.36 | 20.83 | 39.18 | 70.24 | 14.7 | <0.001 |
| Wed | Sun | 65 | 5.66 | 49.5 | 80.5 | 17.5 | <0.001 |
| Thu | Fri | 1.23 | -11.84 | -14.27 | 16.73 | 0.33 | 0.9 |
| Thu | Sat | 54.63 | 8.48 | 38.46 | 69.49 | 14.52 | <0.001 |
| Thu | Sun | 64.27 | -6.69 | 48.79 | 79.75 | 17.32 | <0.001 |
| Fri | Sat | 53.4 | 20.32 | 37.26 | 68.24 | 14.21 | <0.001 |
| Fri | Sun | 63.04 | 5.15 | 47.59 | 78.5 | 17.02 | <0.001 |
| Sat | Sun | -14.42 | -15.17 | -5.18 | 25.76 | 2.78 | 0.44 |
| **Sleep duration** | | | | | | | |
| Mon | Tue | -4.35 | 1.33 | -15.08 | 23.78 | 0.93 | 0.9 |
| Mon | Wed | -0.87 | -3.82 | -18.56 | 20.31 | 0.19 | 0.9 |
| Mon | Thu | -0.1 | -0.08 | -19.31 | 19.52 | 0.02 | 0.9 |
| Mon | Fri | 28.32 | 16.06 | 8.94 | 47.7 | 6.1 | <0.001 |
| Mon | Sat | 32.07 | 17.06 | 12.92 | 51.72 | 6.95 | <0.001 |
| Mon | Sun | -1.47 | -5.23 | -17.89 | 20.83 | 0.32 | 0.9 |
| Tue | Wed | 3.48 | -5.15 | -16.01 | 22.97 | 0.74 | 0.9 |
| Tue | Thu | 4.25 | -1.41 | -15.22 | 23.71 | 0.91 | 0.9 |
| Tue | Fri | 32.67 | 14.72 | 13.23 | 52.1 | 7.01 | <0.001 |
| Tue | Sat | 36.42 | 15.73 | 17.22 | 56.13 | 7.86 | <0.001 |
| Tue | Sun | 2.88 | -6.57 | -16.53 | 22.29 | 0.62 | 0.9 |
| Wed | Thu | 0.77 | 3.74 | -18.7 | 20.23 | 0.16 | 0.9 |
| Wed | Fri | 29.19 | 19.87 | 9.76 | 48.62 | 6.27 | <0.001 |
| Wed | Sat | 32.94 | 20.88 | 13.74 | 52.65 | 7.12 | <0.001 |
| Wed | Sun | -0.6 | -1.41 | -18.81 | 20.01 | 0.13 | 0.9 |
| Thu | Fri | 28.42 | 16.14 | 9.01 | 47.83 | 6.11 | <0.001 |
| Thu | Sat | 32.17 | 17.14 | 12.99 | 51.86 | 6.96 | <0.001 |
| Thu | Sun | -1.37 | -5.15 | -18.02 | 20.76 | 0.29 | 0.9 |
| Fri | Sat | 3.75 | 1 | -15.4 | 23.41 | 0.86 | 0.9 |
| Fri | Sun | -29.79 | -21.29 | 10.43 | 49.15 | 6.42 | <0.001 |
| Sat | Sun | -33.54 | -22.29 | 14.41 | 53.17 | 7.28 | <0.001 |
| **Time spent home** | | | | | | | |
| Mon | Tue | -31.36 | -0.79 | -11.44 | 74.15 | 3.06 | 0.32 |
| Mon | Wed | -15.35 | 3.29 | -27.44 | 58.15 | 1.5 | 0.9 |
| Mon | Thu | -37.73 | -4.33 | -5.01 | 80.48 | 3.68 | 0.12 |
| Mon | Fri | -21.43 | 6.64 | -21.25 | 64.1 | 2.1 | 0.73 |
| Mon | Sat | 16.09 | -3.61 | -26.63 | 58.81 | 1.57 | 0.9 |
| Mon | Sun | 57.96 | 1.06 | 15.33 | 100.59 | 5.67 | <0.001 |
| Tue | Wed | 16.01 | 4.08 | -26.91 | 58.92 | 1.56 | 0.9 |
| Tue | Thu | -6.37 | -3.54 | -36.49 | 49.24 | 0.62 | 0.9 |
| Tue | Fri | 9.93 | 7.43 | -32.87 | 52.72 | 0.97 | 0.9 |
| Tue | Sat | 47.45 | -2.82 | 4.6 | 90.29 | 4.62 | 0.02 |
| Tue | Sun | 89.32 | 1.85 | 46.57 | 132.06 | 8.72 | <0.001 |
| Wed | Thu | -22.38 | -7.62 | -20.49 | 65.25 | 2.18 | 0.69 |
| Wed | Fri | -6.08 | 3.35 | -36.72 | 48.87 | 0.59 | 0.9 |
| Wed | Sat | 31.44 | -6.9 | -11.4 | 74.28 | 3.06 | 0.31 |
| Wed | Sun | 73.31 | -2.23 | 30.56 | 116.06 | 7.16 | <0.001 |
| Thu | Fri | 16.3 | 10.97 | -26.44 | 59.05 | 1.59 | 0.9 |
| Thu | Sat | 53.82 | 0.72 | 11.03 | 96.61 | 5.25 | <0.001 |
| Thu | Sun | 95.69 | 5.39 | 52.99 | 138.39 | 9.35 | <0.001 |
| Fri | Sat | 37.52 | -10.25 | -5.21 | 80.24 | 3.66 | 0.13 |
| Fri | Sun | 79.39 | -5.58 | 36.76 | 122.01 | 7.77 | <0.001 |
| Sat | Sun | 41.87 | 4.67 | -0.8 | 84.54 | 4.09 | 0.06 |

Table S2:Descriptive analysis of seasonal weekdays subgroups for sleep parameters and time spent at home

| **Sleep time** | | | |  | | |  | | |  | |  | | **99% Confidence Interval for Mean** | | | | | |
| --- | --- | --- | --- | --- | --- | --- | --- | --- | --- | --- | --- | --- | --- | --- | --- | --- | --- | --- | --- |
|  |  |  |  | **N** | | | **Mean** | | | **Std. Dev** | | **Std. Err** | | **Lower Bound** | | | | **Upper Bound** | |
| Mon | | Spring | | 478 | | | 23:10 | | | 92.85 | | 4.25 | | 22:59 | | | | 23:21 | |
|  |  | Summer | | 473 | | | 23:05 | | | 79.67 | | 3.66 | | 22:56 | | | | 23:14 | |
|  |  | Fall | | 475 | | | 23:03 | | | 88.98 | | 4.08 | | 22:53 | | | | 23:14 | |
|  |  | Winter | | 471 | | | 23:19 | | | 99.77 | | 4.6 | | 23:07 | | | | 23:31 | |
| Tue | | Spring | | 477 | | | 23:24 | | | 96.74 | | 4.43 | | 23:13 | | | | 23:36 | |
|  |  | Summer | | 474 | | | 23:11 | | | 98.7 | | 4.53 | | 22:59 | | | | 23:23 | |
|  |  | Fall | | 474 | | | 23:15 | | | 92.97 | | 4.27 | | 23:04 | | | | 23:26 | |
|  |  | Winter | | 468 | | | 23:23 | | | 105.58 | | 4.88 | | 23:11 | | | | 23:36 | |
| Wed | | Spring | | 473 | | | 23:11 | | | 88.02 | | 4.05 | | 23:01 | | | | 23:21 | |
|  |  | Summer | | 472 | | | 23:12 | | | 88.53 | | 4.07 | | 23:01 | | | | 23:22 | |
|  |  | Fall | | 478 | | | 23:09 | | | 92.13 | | 4.21 | | 22:58 | | | | 23:20 | |
|  |  | Winter | | 470 | | | 23:12 | | | 101.15 | | 4.67 | | 23:00 | | | | 23:24 | |
| Thu | | Spring | | 472 | | | 23:15 | | | 93.92 | | 4.32 | | 23:04 | | | | 23:26 | |
|  |  | Summer | | 473 | | | 23:27 | | | 93.28 | | 4.29 | | 23:16 | | | | 23:38 | |
|  |  | Fall | | 477 | | | 23:10 | | | 86.74 | | 3.97 | | 23:00 | | | | 23:20 | |
|  |  | Winter | | 470 | | | 23:12 | | | 96.3 | | 4.44 | | 23:00 | | | | 23:23 | |
| Fri | | Spring | | 478 | | | 23:32 | | | 90.77 | | 4.15 | | 23:22 | | | | 23:43 | |
|  |  | Summer | | 475 | | | 23:34 | | | 83.44 | | 3.83 | | 23:24 | | | | 23:44 | |
|  |  | Fall | | 474 | | | 23:29 | | | 86.7 | | 3.98 | | 23:19 | | | | 23:39 | |
|  |  | Winter | | 471 | | | 23:36 | | | 91.19 | | 4.2 | | 23:25 | | | | 23:47 | |
| Sat | | Spring | | 475 | | | 23:42 | | | 83.18 | | 3.82 | | 23:32 | | | | 23:52 | |
|  |  | Summer | | 472 | | | 23:43 | | | 90.51 | | 4.17 | | 23:32 | | | | 23:54 | |
|  |  | Fall | | 473 | | | 23:33 | | | 84.43 | | 3.88 | | 23:23 | | | | 23:43 | |
|  |  | Winter | | 469 | | | 23:48 | | | 100.62 | | 4.65 | | 23:36 | | | | 00:00 | |
| Sun | | Spring | | 477 | | | 23:17 | | | 90.26 | | 4.13 | | 23:07 | | | | 23:28 | |
|  |  | Summer | | 474 | | | 23:21 | | | 95.19 | | 4.37 | | 23:10 | | | | 23:32 | |
|  |  | Fall | | 475 | | | 23:08 | | | 91.22 | | 4.19 | | 22:57 | | | | 23:18 | |
|  |  | Winter | | 469 | | | 23:16 | | | 92.03 | | 4.25 | | 23:05 | | | | 23:27 | |
| **Wake-up time** | | | |  | | |  | | |  | |  | | | **99% Confidence Interval for Mean** | | | | |
|  |  |  |  | **N** | | | **Mean** | | | **Std. Dev** | | **Std. Err** | | | **Lower Bound** | | | **Upper Bound** | |
| Mon | | Spring | | 478 | | | 5:38 | | | 79.2 | | 3.62 | | | 5:28 | | | 5:47 | |
|  |  | Summer | | 473 | | | 5:54 | | | 76.62 | | 3.52 | | | 5:45 | | | 6:03 | |
|  |  | Fall | | 475 | | | 5:35 | | | 77.63 | | 3.56 | | | 5:26 | | | 5:44 | |
|  |  | Winter | | 471 | | | 5:36 | | | 78.04 | | 3.6 | | | 5:27 | | | 5:45 | |
| Tue | | Spring | | 477 | | | 5:31 | | | 76.49 | | 3.5 | | | 5:22 | | | 5:40 | |
|  |  | Summer | | 474 | | | 5:41 | | | 74.06 | | 3.4 | | | 5:33 | | | 5:50 | |
|  |  | Fall | | 474 | | | 5:30 | | | 90.72 | | 4.17 | | | 5:20 | | | 5:41 | |
|  |  | Winter | | 468 | | | 5:36 | | | 110.23 | | 5.1 | | | 5:23 | | | 5:49 | |
| Wed | | Spring | | 473 | | | 5:38 | | | 77.07 | | 3.54 | | | 5:29 | | | 5:47 | |
|  |  | Summer | | 472 | | | 5:50 | | | 76.57 | | 3.52 | | | 5:41 | | | 5:59 | |
|  |  | Fall | | 478 | | | 5:36 | | | 78.46 | | 3.59 | | | 5:27 | | | 5:46 | |
|  |  | Winter | | 470 | | | 5:41 | | | 90.42 | | 4.17 | | | 5:30 | | | 5:51 | |
| Thu | | Spring | | 472 | | | 5:33 | | | 83.5 | | 3.84 | | | 5:23 | | | 5:43 | |
|  |  | Summer | | 473 | | | 5:45 | | | 76.66 | | 3.52 | | | 5:36 | | | 5:54 | |
|  |  | Fall | | 477 | | | 5:42 | | | 73.7 | | 3.37 | | | 5:33 | | | 5:51 | |
|  |  | Winter | | 470 | | | 5:32 | | | 80.5 | | 3.71 | | | 5:22 | | | 5:41 | |
| Fri | | Spring | | 478 | | | 5:39 | | | 91.98 | | 4.21 | | | 5:28 | | | 5:50 | |
|  |  | Summer | | 475 | | | 5:55 | | | 86.24 | | 3.96 | | | 5:45 | | | 6:05 | |
|  |  | Fall | | 474 | | | 5:41 | | | 82.43 | | 3.79 | | | 5:31 | | | 5:51 | |
|  |  | Winter | | 471 | | | 5:31 | | | 82.34 | | 3.79 | | | 5:21 | | | 5:41 | |
| Sat | | Spring | | 475 | | | 6:12 | | | 82.38 | | 3.78 | | | 6:02 | | | 6:22 | |
|  |  | Summer | | 472 | | | 6:27 | | | 92.66 | | 4.26 | | | 6:16 | | | 6:38 | |
|  |  | Fall | | 473 | | | 6:19 | | | 83.81 | | 3.85 | | | 6:09 | | | 6:29 | |
|  |  | Winter | | 469 | | | 6:16 | | | 102.3 | | 4.72 | | | 6:03 | | | 6:28 | |
| Sun | | Spring | | 477 | | | 6:21 | | | 99.01 | | 4.53 | | | 6:09 | | | 6:33 | |
|  |  | Summer | | 474 | | | 6:36 | | | 83.32 | | 3.83 | | | 6:27 | | | 6:46 | |
|  |  | Fall | | 475 | | | 6:31 | | | 82.07 | | 3.77 | | | 6:21 | | | 6:40 | |
|  |  | Winter | | 469 | | | 6:35 | | | 91.84 | | 4.24 | | | 6:24 | | | 6:46 | |
| **Sleep duration** | | | |  | |  | | |  | | |  | | **99% Confidence Interval for Mean** | | | | | |
|  |  |  |  | **N** | | **Mean** | | | **Std. Dev** | | | **Std. Err** | | **Lower Bound** | | **Upper Bound** | | | |
| Mon | | Spring | | 478 | | 379.98 | | | 100.47 | | | 4.6 | | 368.14 | | | | | 391.82 |
|  |  | Summer | | 473 | | 395.88 | | | 98.07 | | | 4.51 | | 384.26 | | | | | 407.49 |
|  |  | Fall | | 475 | | 385.56 | | | 102.43 | | | 4.7 | | 373.45 | | | | | 397.66 |
|  |  | Winter | | 471 | | 371.88 | | | 101.79 | | | 4.69 | | 359.8 | | | | | 383.96 |
| Tue | | Spring | | 477 | | 374.24 | | | 97.09 | | | 4.45 | | 362.79 | | | | | 385.69 |
|  |  | Summer | | 474 | | 395.78 | | | 99.79 | | | 4.58 | | 383.97 | | | | | 407.58 |
|  |  | Fall | | 474 | | 379.93 | | | 96.65 | | | 4.44 | | 368.5 | | | | | 391.37 |
|  |  | Winter | | 468 | | 375.02 | | | 107.84 | | | 4.98 | | 362.18 | | | | | 387.86 |
| Wed | | Spring | | 473 | | 378.11 | | | 96.37 | | | 4.43 | | 366.7 | | | | | 389.53 |
|  |  | Summer | | 472 | | 392.94 | | | 97.25 | | | 4.48 | | 381.41 | | | | | 404.47 |
|  |  | Fall | | 478 | | 393.1 | | | 95.68 | | | 4.38 | | 381.82 | | | | | 404.37 |
|  |  | Winter | | 470 | | 379.34 | | | 106.72 | | | 4.92 | | 366.66 | | | | | 392.02 |
| Thu | | Spring | | 472 | | 382.1 | | | 99.13 | | | 4.56 | | 370.35 | | | | | 393.86 |
|  |  | Summer | | 473 | | 384.39 | | | 97.04 | | | 4.46 | | 372.9 | | | | | 395.89 |
|  |  | Fall | | 477 | | 388.49 | | | 95.5 | | | 4.37 | | 377.23 | | | | | 399.76 |
|  |  | Winter | | 470 | | 378.24 | | | 103.41 | | | 4.77 | | 365.95 | | | | | 390.53 |
| Fri | | Spring | | 478 | | 400.38 | | | 109.41 | | | 5 | | 387.49 | | | | | 413.27 |
|  |  | Summer | | 475 | | 410.01 | | | 104.08 | | | 4.78 | | 397.71 | | | | | 422.31 |
|  |  | Fall | | 474 | | 410.28 | | | 107.94 | | | 4.96 | | 397.51 | | | | | 423.05 |
|  |  | Winter | | 471 | | 396.69 | | | 115.95 | | | 5.34 | | 382.93 | | | | | 410.45 |
| Sat | | Spring | | 475 | | 395.96 | | | 110.61 | | | 5.08 | | 382.89 | | | | | 409.03 |
|  |  | Summer | | 472 | | 413.07 | | | 107.67 | | | 4.96 | | 400.31 | | | | | 425.84 |
|  |  | Fall | | 473 | | 418.16 | | | 103.7 | | | 4.77 | | 405.87 | | | | | 430.44 |
|  |  | Winter | | 469 | | 404.58 | | | 115.2 | | | 5.32 | | 390.88 | | | | | 418.28 |
| Sun | | Spring | | 477 | | 379.36 | | | 99.33 | | | 4.55 | | 367.64 | | | | | 391.07 |
|  |  | Summer | | 474 | | 389.49 | | | 98.55 | | | 4.53 | | 377.83 | | | | | 401.15 |
|  |  | Fall | | 475 | | 386.15 | | | 99.1 | | | 4.55 | | 374.43 | | | | | 397.86 |
|  |  | Winter | | 469 | | 379.76 | | | 103.03 | | | 4.76 | | 367.51 | | | | | 392.02 |
| **Time spent home** | | |  | |  | | |  | | |  | | **99% Confidence Interval for Mean** | | | | | | |
|  |  |  | **N** | | **Mean** | | | **Std. Dev** | | | **Std. Err** | | **Lower Bound** | | | | **Upper Bound** | | |
| Mon | Spring | | 478 | | 545.63 | | | 211.28 | | | 9.66 | | 520.73 | | | | 570.52 | | |
|  | Summer | | 473 | | 526.71 | | | 216.16 | | | 9.94 | | 501.11 | | | | 552.31 | | |
|  | Fall | | 475 | | 547.04 | | | 208.71 | | | 9.58 | | 522.37 | | | | 571.71 | | |
|  | Winter | | 471 | | 525.11 | | | 197.27 | | | 9.09 | | 501.7 | | | | 548.53 | | |
| Tue | Spring | | 477 | | 503.32 | | | 198.37 | | | 9.08 | | 479.93 | | | | 526.72 | | |
|  | Summer | | 474 | | 503.61 | | | 216.83 | | | 9.96 | | 477.96 | | | | 529.27 | | |
|  | Fall | | 474 | | 506.62 | | | 200.29 | | | 9.2 | | 482.92 | | | | 530.31 | | |
|  | Winter | | 468 | | 538.12 | | | 208.77 | | | 9.65 | | 513.26 | | | | 562.98 | | |
| Wed | Spring | | 473 | | 531.5 | | | 209.86 | | | 9.65 | | 506.65 | | | | 556.36 | | |
|  | Summer | | 472 | | 511.08 | | | 212.16 | | | 9.77 | | 485.92 | | | | 536.23 | | |
|  | Fall | | 478 | | 524.44 | | | 207.12 | | | 9.47 | | 500.04 | | | | 548.85 | | |
|  | Winter | | 470 | | 544.73 | | | 213.61 | | | 9.85 | | 519.35 | | | | 570.11 | | |
| Thu | Spring | | 472 | | 532.73 | | | 202.9 | | | 9.34 | | 508.68 | | | | 556.79 | | |
|  | Summer | | 473 | | 458.55 | | | 199.41 | | | 9.17 | | 434.94 | | | | 482.17 | | |
|  | Fall | | 477 | | 519.5 | | | 202.06 | | | 9.25 | | 495.67 | | | | 543.34 | | |
|  | Winter | | 470 | | 538.39 | | | 204.29 | | | 9.42 | | 514.12 | | | | 562.67 | | |
| Fri | Spring | | 478 | | 524.58 | | | 207.88 | | | 9.51 | | 500.09 | | | | 549.07 | | |
|  | Summer | | 475 | | 498.61 | | | 215.74 | | | 9.9 | | 473.12 | | | | 524.11 | | |
|  | Fall | | 474 | | 531.85 | | | 207.81 | | | 9.54 | | 507.26 | | | | 556.43 | | |
|  | Winter | | 471 | | 556.83 | | | 217.32 | | | 10.01 | | 531.03 | | | | 582.62 | | |
| Sat | Spring | | 475 | | 553.34 | | | 202.77 | | | 9.3 | | 529.38 | | | | 577.3 | | |
|  | Summer | | 472 | | 514.19 | | | 204.15 | | | 9.4 | | 489.98 | | | | 538.39 | | |
|  | Fall | | 473 | | 575.92 | | | 198.34 | | | 9.12 | | 552.43 | | | | 599.41 | | |
|  | Winter | | 469 | | 596.49 | | | 211.83 | | | 9.78 | | 571.29 | | | | 621.68 | | |
| Sun | Spring | | 477 | | 591.57 | | | 201.11 | | | 9.21 | | 567.86 | | | | 615.29 | | |
|  | Summer | | 474 | | 554.07 | | | 206.59 | | | 9.49 | | 529.63 | | | | 578.51 | | |
|  | Fall | | 475 | | 607.47 | | | 206.75 | | | 9.49 | | 583.04 | | | | 631.91 | | |
|  | Winter | | 469 | | 626.08 | | | 213.83 | | | 9.87 | | 600.65 | | | | 651.52 | | |

Table S3. Evaluating the homogeneity of variances in different weekdays for all season groups using the Levene test

| **Indicator** | **Weekday (through seasons)** | **P-value** |
| --- | --- | --- |
| Wake up | Mon | 0.97 |
|  | Tue | 0.02 |
|  | Wed | 0.43 |
|  | Thu | 0.20 |
|  | Fri | 0.38 |
|  | Sat | 0.28 |
|  | Sun | 0.36 |
| Sleep time | Mon | 0.03 |
|  | Tue | 0.03 |
|  | Wed | 0.31 |
|  | Thu | 0.18 |
|  | Fri | 0.41 |
|  | Sat | <0.001 |
|  | Sun | 0.79 |
| Sleep duration | Mon | 0.76 |
|  | Tue | 0.10 |
|  | Wed | 0.06 |
|  | Thu | 0.08 |
|  | Fri | 0.04 |
|  | Sat | 0.12 |
|  | Sun | 0.62 |
| Time spent home | Mon | 0.36 |
|  | Tue | 0.18 |
|  | Wed | 0.88 |
|  | Thu | 0.86 |
|  | Fri | 0.64 |
|  | Sat | 0.45 |
|  | Sun | 0.53 |

Table S4: The Tukey post hoc test compare sleep parameters and time spent at home with respect to different seasonal weekday pairs

| group1 | group2 | Diff | Std_Diff | Lower | Upper | q-value | p-value |
| --- | --- | --- | --- | --- | --- | --- | --- |
| **Sleep time** | | | | | | | |
| ( winter , Mon ) | ( spring , Mon ) | -8.2 | -6.24 | -14.85 | 31.25 | 1.87 | 0.9 |
| ( winter , Mon ) | ( summer , Mon ) | -12.46 | -19.46 | -10.67 | 35.6 | 2.83 | 0.9 |
| ( winter , Mon ) | ( fall , Mon ) | -16.21 | -10.92 | -6.85 | 39.27 | 3.69 | 0.64 |
| ( spring , Mon ) | ( summer , Mon ) | -4.27 | -13.21 | -18.82 | 27.35 | 0.97 | 0.9 |
| ( spring , Mon ) | ( fall , Mon ) | -8.01 | -4.68 | -15 | 31.03 | 1.83 | 0.9 |
| ( summer , Mon ) | ( fall , Mon ) | -3.75 | 8.53 | -19.35 | 26.85 | 0.85 | 0.9 |
| ( winter , Tue ) | ( spring , Tue ) | 2.46 | -9.4 | -20.67 | 25.58 | 0.56 | 0.9 |
| ( winter , Tue ) | ( summer , Tue ) | -7.6 | 2.26 | -15.57 | 30.76 | 1.72 | 0.9 |
| ( winter , Tue ) | ( fall , Tue ) | -3.62 | -11.11 | -19.55 | 26.78 | 0.82 | 0.9 |
| ( spring , Tue ) | ( summer , Tue ) | -10.05 | 11.65 | -13.01 | 33.12 | 2.29 | 0.9 |
| ( spring , Tue ) | ( fall , Tue ) | -6.07 | -1.71 | -16.99 | 29.14 | 1.38 | 0.9 |
| ( summer , Tue ) | ( fall , Tue ) | 3.98 | -13.36 | -19.12 | 27.08 | 0.9 | 0.9 |
| ( winter , Wed ) | ( spring , Wed ) | 0.81 | -10.97 | -22.35 | 23.97 | 0.18 | 0.9 |
| ( winter , Wed ) | ( summer , Wed ) | 3.13 | -7.07 | -20.05 | 26.31 | 0.71 | 0.9 |
| ( winter , Wed ) | ( fall , Wed ) | -1.22 | -8.57 | -21.89 | 24.34 | 0.28 | 0.9 |
| ( spring , Wed ) | ( summer , Wed ) | 2.32 | 3.9 | -20.79 | 25.43 | 0.53 | 0.9 |
| ( spring , Wed ) | ( fall , Wed ) | -2.03 | 2.4 | -21.02 | 25.08 | 0.46 | 0.9 |
| ( summer , Wed ) | ( fall , Wed ) | -4.35 | -1.49 | -18.71 | 27.42 | 0.99 | 0.9 |
| ( winter , Thu ) | ( spring , Thu ) | 3.75 | -2.2 | -19.41 | 26.92 | 0.85 | 0.9 |
| ( winter , Thu ) | ( summer , Thu ) | 18.16 | 1.5 | -5.01 | 41.34 | 4.12 | 0.43 |
| ( winter , Thu ) | ( fall , Thu ) | -2.22 | -11.05 | -20.92 | 25.36 | 0.51 | 0.9 |
| ( spring , Thu ) | ( summer , Thu ) | 14.41 | 3.7 | -8.68 | 37.5 | 3.28 | 0.83 |
| ( spring , Thu ) | ( fall , Thu ) | -5.98 | -8.86 | -17.07 | 29.03 | 1.36 | 0.9 |
| ( summer , Thu ) | ( fall , Thu ) | -20.39 | -12.55 | -2.68 | 43.45 | 4.64 | 0.18 |
| ( winter , Fri ) | ( spring , Fri ) | -4.78 | -1 | -18.32 | 27.88 | 1.09 | 0.9 |
| ( winter , Fri ) | ( summer , Fri ) | -1.01 | -4.37 | -22.15 | 24.16 | 0.23 | 0.9 |
| ( winter , Fri ) | ( fall , Fri ) | -4.44 | -4.43 | -18.7 | 27.58 | 1.01 | 0.9 |
| ( spring , Fri ) | ( summer , Fri ) | 3.78 | -3.37 | -19.27 | 26.83 | 0.86 | 0.9 |
| ( spring , Fri ) | ( fall , Fri ) | 0.34 | -3.42 | -22.7 | 23.38 | 0.08 | 0.9 |
| ( summer , Fri ) | ( fall , Fri ) | -3.43 | -0.06 | -19.65 | 26.52 | 0.78 | 0.9 |
| ( winter , Sat ) | ( spring , Sat ) | -4.18 | -13.68 | -18.97 | 27.33 | 0.95 | 0.9 |
| ( winter , Sat ) | ( summer , Sat ) | -3.82 | -7.7 | -19.36 | 26.99 | 0.87 | 0.9 |
| ( winter , Sat ) | ( fall , Sat ) | -16.16 | -14.47 | -7.01 | 39.32 | 3.66 | 0.65 |
| ( spring , Sat ) | ( summer , Sat ) | 0.37 | 5.98 | -22.76 | 23.49 | 0.08 | 0.9 |
| ( spring , Sat ) | ( fall , Sat ) | -11.97 | -0.79 | -11.14 | 35.09 | 2.72 | 0.9 |
| ( summer , Sat ) | ( fall , Sat ) | -12.34 | -6.77 | -10.8 | 35.48 | 2.8 | 0.9 |
| ( winter , Sun ) | ( spring , Sun ) | 0.65 | -4.53 | -22.47 | 23.76 | 0.15 | 0.9 |
| ( winter , Sun ) | ( summer , Sun ) | 0.32 | 13.24 | -22.82 | 23.46 | 0.07 | 0.9 |
| ( winter , Sun ) | ( fall , Sun ) | -9.01 | -2.7 | -14.09 | 32.12 | 2.05 | 0.9 |
| ( spring , Sun ) | ( summer , Sun ) | -0.33 | 17.77 | -22.75 | 23.4 | 0.07 | 0.9 |
| ( spring , Sun ) | ( fall , Sun ) | -9.66 | 1.83 | -13.38 | 32.7 | 2.2 | 0.9 |
| ( summer , Sun ) | ( fall , Sun ) | -9.33 | -15.94 | -13.73 | 32.4 | 2.13 | 0.9 |
| **Wakeup time** | | | | | | | |
| ( winter , Mon ) | ( spring , Mon ) | 1.71 | -0.24 | -18.86 | 22.29 | 0.44 | 0.9 |
| ( winter , Mon ) | ( summer , Mon ) | 18.3 | 0.45 | -2.35 | 38.95 | 4.66 | 0.18 |
| ( winter , Mon ) | ( fall , Mon ) | -2.08 | -0.88 | -18.51 | 22.66 | 0.53 | 0.9 |
| ( spring , Mon ) | ( summer , Mon ) | 16.59 | 0.69 | -4.02 | 37.19 | 4.23 | 0.36 |
| ( spring , Mon ) | ( fall , Mon ) | -3.79 | -0.64 | -16.75 | 24.33 | 0.97 | 0.9 |
| ( summer , Mon ) | ( fall , Mon ) | -20.37 | -1.33 | -0.24 | 40.99 | 5.19 | 0.06 |
| ( winter , Tue ) | ( spring , Tue ) | -7.6 | -37.49 | -13.04 | 28.24 | 1.93 | 0.9 |
| ( winter , Tue ) | ( summer , Tue ) | 2.34 | -37.38 | -18.34 | 23.02 | 0.59 | 0.9 |
| ( winter , Tue ) | ( fall , Tue ) | -7.52 | -18.83 | -13.16 | 28.2 | 1.91 | 0.9 |
| ( spring , Tue ) | ( summer , Tue ) | 9.94 | 0.11 | -10.65 | 30.53 | 2.54 | 0.9 |
| ( spring , Tue ) | ( fall , Tue ) | 0.08 | 18.66 | -20.51 | 20.67 | 0.02 | 0.9 |
| ( summer , Tue ) | ( fall , Tue ) | -9.86 | 18.55 | -10.76 | 30.48 | 2.51 | 0.9 |
| ( winter , Wed ) | ( spring , Wed ) | -3.26 | -15.09 | -17.42 | 23.93 | 0.83 | 0.9 |
| ( winter , Wed ) | ( summer , Wed ) | 12.41 | -12.69 | -8.27 | 33.1 | 3.15 | 0.89 |
| ( winter , Wed ) | ( fall , Wed ) | -3.52 | -12.54 | -17.11 | 24.15 | 0.9 | 0.9 |
| ( spring , Wed ) | ( summer , Wed ) | 15.67 | 2.4 | -4.96 | 36.3 | 3.99 | 0.49 |
| ( spring , Wed ) | ( fall , Wed ) | -0.26 | 2.55 | -20.31 | 20.83 | 0.07 | 0.9 |
| ( summer , Wed ) | ( fall , Wed ) | -15.93 | 0.15 | -4.65 | 36.52 | 4.07 | 0.46 |
| ( winter , Thu ) | ( spring , Thu ) | 3.17 | 3.45 | -17.5 | 23.85 | 0.81 | 0.9 |
| ( winter , Thu ) | ( summer , Thu ) | 12.81 | -1.76 | -7.88 | 33.49 | 3.25 | 0.85 |
| ( winter , Thu ) | ( fall , Thu ) | 9.73 | -4.76 | -10.92 | 30.39 | 2.48 | 0.9 |
| ( spring , Thu ) | ( summer , Thu ) | 9.63 | -5.21 | -10.98 | 30.24 | 2.46 | 0.9 |
| ( spring , Thu ) | ( fall , Thu ) | 6.56 | -8.21 | -14.01 | 27.13 | 1.68 | 0.9 |
| ( summer , Thu ) | ( fall , Thu ) | -3.07 | -3 | -17.51 | 23.66 | 0.78 | 0.9 |
| ( winter , Fri ) | ( spring , Fri ) | 8.74 | 8.35 | -11.88 | 29.36 | 2.23 | 0.9 |
| ( winter , Fri ) | ( summer , Fri ) | 27.06 | 4.93 | 6.4 | 47.73 | 6.88 | 0 ** |
| ( winter , Fri ) | ( fall , Fri ) | 6.81 | -12.87 | -13.85 | 27.46 | 1.73 | 0.9 |
| ( spring , Fri ) | ( summer , Fri ) | 18.32 | -3.42 | -2.25 | 38.9 | 4.68 | 0.17 |
| ( spring , Fri ) | ( fall , Fri ) | -1.93 | -21.22 | -18.63 | 22.49 | 0.49 | 0.9 |
| ( summer , Fri ) | ( fall , Fri ) | -20.26 | -17.79 | -0.35 | 40.86 | 5.16 | 0.06 |
| ( winter , Sat ) | ( spring , Sat ) | -4.15 | -20.03 | -16.52 | 24.81 | 1.05 | 0.9 |
| ( winter , Sat ) | ( summer , Sat ) | 12.32 | -7.96 | -8.37 | 33.01 | 3.13 | 0.9 |
| ( winter , Sat ) | ( fall , Sat ) | 2.97 | -19.53 | -17.71 | 23.64 | 0.75 | 0.9 |
| ( spring , Sat ) | ( summer , Sat ) | 16.47 | 12.07 | -4.17 | 37.11 | 4.19 | 0.39 |
| ( spring , Sat ) | ( fall , Sat ) | 7.11 | 0.5 | -13.52 | 27.74 | 1.81 | 0.9 |
| ( summer , Sat ) | ( fall , Sat ) | -9.35 | -11.58 | -11.3 | 30.01 | 2.38 | 0.9 |
| ( winter , Sun ) | ( spring , Sun ) | -15.08 | 5.54 | -5.56 | 35.71 | 3.84 | 0.57 |
| ( winter , Sun ) | ( summer , Sun ) | -0.07 | -7.47 | -20.59 | 20.72 | 0.02 | 0.9 |
| ( winter , Sun ) | ( fall , Sun ) | -7.21 | -9.27 | -13.41 | 27.82 | 1.84 | 0.9 |
| ( spring , Sun ) | ( summer , Sun ) | 15.01 | -13.01 | -5.59 | 35.6 | 3.83 | 0.57 |
| ( spring , Sun ) | ( fall , Sun ) | 7.87 | -14.81 | -12.69 | 28.43 | 2.01 | 0.9 |
| ( summer , Sun ) | ( fall , Sun ) | -7.14 | -1.8 | -13.45 | 27.72 | 1.82 | 0.9 |
| **Sleep duration** | | | | | | | |
| ( winter , Mon ) | ( spring , Mon ) | 0.1 | -0.03 | -0.32 | 0.51 | 1.2 | 0.9 |
| ( winter , Mon ) | ( summer , Mon ) | 0.32 | -0.08 | -0.1 | 0.74 | 4.01 | 0.48 |
| ( winter , Mon ) | ( fall , Mon ) | 0.2 | 0.04 | -0.22 | 0.62 | 2.52 | 0.9 |
| ( spring , Mon ) | ( summer , Mon ) | 0.22 | -0.05 | -0.19 | 0.64 | 2.82 | 0.9 |
| ( spring , Mon ) | ( fall , Mon ) | 0.1 | 0.07 | -0.31 | 0.52 | 1.32 | 0.9 |
| ( summer , Mon ) | ( fall , Mon ) | -0.12 | 0.12 | -0.3 | 0.54 | 1.5 | 0.9 |
| ( winter , Tue ) | ( spring , Tue ) | -0.05 | -0.19 | -0.37 | 0.46 | 0.58 | 0.9 |
| ( winter , Tue ) | ( summer , Tue ) | 0.31 | -0.12 | -0.11 | 0.73 | 3.94 | 0.52 |
| ( winter , Tue ) | ( fall , Tue ) | 0.04 | -0.23 | -0.38 | 0.45 | 0.45 | 0.9 |
| ( spring , Tue ) | ( summer , Tue ) | 0.36 | 0.07 | -0.06 | 0.78 | 4.53 | 0.22 |
| ( spring , Tue ) | ( fall , Tue ) | 0.08 | -0.03 | -0.34 | 0.5 | 1.03 | 0.9 |
| ( summer , Tue ) | ( fall , Tue ) | -0.28 | -0.11 | -0.14 | 0.7 | 3.5 | 0.73 |
| ( winter , Wed ) | ( spring , Wed ) | -0.01 | -0.17 | -0.41 | 0.43 | 0.17 | 0.9 |
| ( winter , Wed ) | ( summer , Wed ) | 0.18 | -0.12 | -0.24 | 0.6 | 2.22 | 0.9 |
| ( winter , Wed ) | ( fall , Wed ) | 0.19 | -0.18 | -0.23 | 0.61 | 2.36 | 0.9 |
| ( spring , Wed ) | ( summer , Wed ) | 0.19 | 0.04 | -0.23 | 0.61 | 2.39 | 0.9 |
| ( spring , Wed ) | ( fall , Wed ) | 0.2 | -0.01 | -0.22 | 0.62 | 2.53 | 0.9 |
| ( summer , Wed ) | ( fall , Wed ) | 0.01 | -0.05 | -0.41 | 0.43 | 0.13 | 0.9 |
| ( winter , Thu ) | ( spring , Thu ) | 0.06 | -0.09 | -0.36 | 0.48 | 0.79 | 0.9 |
| ( winter , Thu ) | ( summer , Thu ) | 0.12 | -0.14 | -0.3 | 0.54 | 1.45 | 0.9 |
| ( winter , Thu ) | ( fall , Thu ) | 0.18 | -0.17 | -0.24 | 0.6 | 2.28 | 0.9 |
| ( spring , Thu ) | ( summer , Thu ) | 0.05 | -0.05 | -0.37 | 0.47 | 0.66 | 0.9 |
| ( spring , Thu ) | ( fall , Thu ) | 0.12 | -0.07 | -0.3 | 0.54 | 1.49 | 0.9 |
| ( summer , Thu ) | ( fall , Thu ) | 0.07 | -0.02 | -0.35 | 0.48 | 0.83 | 0.9 |
| ( winter , Fri ) | ( spring , Fri ) | 0.07 | -0.08 | -0.35 | 0.48 | 0.84 | 0.9 |
| ( winter , Fri ) | ( summer , Fri ) | 0.21 | -0.14 | -0.21 | 0.63 | 2.65 | 0.9 |
| ( winter , Fri ) | ( fall , Fri ) | 0.16 | -0.12 | -0.25 | 0.58 | 2.07 | 0.9 |
| ( spring , Fri ) | ( summer , Fri ) | 0.14 | -0.06 | -0.27 | 0.56 | 1.82 | 0.9 |
| ( spring , Fri ) | ( fall , Fri ) | 0.1 | -0.04 | -0.32 | 0.52 | 1.24 | 0.9 |
| ( summer , Fri ) | ( fall , Fri ) | -0.05 | 0.02 | -0.37 | 0.46 | 0.59 | 0.9 |
| ( winter , Sat ) | ( spring , Sat ) | -0.19 | -0.08 | -0.23 | 0.61 | 2.34 | 0.9 |
| ( winter , Sat ) | ( summer , Sat ) | 0.1 | -0.09 | -0.32 | 0.52 | 1.27 | 0.9 |
| ( winter , Sat ) | ( fall , Sat ) | 0.19 | -0.15 | -0.23 | 0.6 | 2.32 | 0.9 |
| ( spring , Sat ) | ( summer , Sat ) | 0.29 | -0.01 | -0.13 | 0.71 | 3.61 | 0.68 |
| ( spring , Sat ) | ( fall , Sat ) | 0.37 | -0.07 | -0.05 | 0.79 | 4.67 | 0.18 |
| ( summer , Sat ) | ( fall , Sat ) | 0.08 | -0.06 | -0.33 | 0.5 | 1.05 | 0.9 |
| ( winter , Sun ) | ( spring , Sun ) | -0.02 | -0.08 | -0.4 | 0.43 | 0.19 | 0.9 |
| ( winter , Sun ) | ( summer , Sun ) | 0.13 | -0.09 | -0.29 | 0.55 | 1.65 | 0.9 |
| ( winter , Sun ) | ( fall , Sun ) | 0.1 | -0.09 | -0.32 | 0.52 | 1.25 | 0.9 |
| ( spring , Sun ) | ( summer , Sun ) | 0.15 | -0.01 | -0.27 | 0.56 | 1.84 | 0.9 |
| ( spring , Sun ) | ( fall , Sun ) | 0.11 | -0.01 | -0.3 | 0.53 | 1.45 | 0.9 |
| ( summer , Sun ) | ( fall , Sun ) | 0.1 | 0 | -0.39 | 0.45 | 0.4 | 0.9 |
| **Time spent home** | | | | | | | |
| ( winter , Mon ) | ( spring , Mon ) | 24.18 | 10.42 | -26.14 | 74.51 | 2.53 | 0.9 |
| ( winter , Mon ) | ( summer , Mon ) | 4.41 | 16.12 | -46.11 | 54.92 | 0.46 | 0.9 |
| ( winter , Mon ) | ( fall , Mon ) | 24.54 | 10.03 | -25.81 | 74.89 | 2.56 | 0.9 |
| ( spring , Mon ) | ( summer , Mon ) | -19.78 | 5.7 | -30.63 | 70.18 | 2.06 | 0.9 |
| ( spring , Mon ) | ( fall , Mon ) | 0.36 | -0.39 | -49.88 | 50.6 | 0.04 | 0.9 |
| ( summer , Mon ) | ( fall , Mon ) | 20.14 | -6.09 | -30.3 | 70.57 | 2.1 | 0.9 |
| ( winter , Tue ) | ( spring , Tue ) | -31.27 | -10.96 | -19.22 | 81.76 | 3.25 | 0.85 |
| ( winter , Tue ) | ( summer , Tue ) | -35.3 | 4.98 | -15.27 | 85.87 | 3.67 | 0.65 |
| ( winter , Tue ) | ( fall , Tue ) | -29.95 | -9.25 | -20.62 | 80.52 | 3.11 | 0.9 |
| ( spring , Tue ) | ( summer , Tue ) | -4.03 | 15.94 | -46.32 | 54.38 | 0.42 | 0.9 |
| ( spring , Tue ) | ( fall , Tue ) | 1.32 | 1.7 | -49.03 | 51.67 | 0.14 | 0.9 |
| ( summer , Tue ) | ( fall , Tue ) | 5.35 | -14.24 | -45.08 | 55.78 | 0.56 | 0.9 |
| ( winter , Wed ) | ( spring , Wed ) | -14.25 | -1.86 | -36.32 | 64.83 | 1.48 | 0.9 |
| ( winter , Wed ) | ( summer , Wed ) | -38.03 | 0.56 | -12.57 | 88.63 | 3.95 | 0.51 |
| ( winter , Wed ) | ( fall , Wed ) | -21.74 | -7.46 | -28.73 | 72.2 | 2.26 | 0.9 |
| ( spring , Wed ) | ( summer , Wed ) | -23.77 | 2.42 | -26.69 | 74.23 | 2.48 | 0.9 |
| ( spring , Wed ) | ( fall , Wed ) | -7.48 | -5.59 | -42.84 | 57.8 | 0.78 | 0.9 |
| ( summer , Wed ) | ( fall , Wed ) | 16.29 | -8.01 | -34.06 | 66.64 | 1.7 | 0.9 |
| ( winter , Thu ) | ( spring , Thu ) | -7.15 | -1.7 | -43.42 | 57.72 | 0.74 | 0.9 |
| ( winter , Thu ) | ( summer , Thu ) | -81.23 | -5.77 | 30.64 | 131.83 | 8.44 | <0.001 |
| ( winter , Thu ) | ( fall , Thu ) | -23.07 | -6.48 | -27.44 | 73.59 | 2.4 | 0.9 |
| ( spring , Thu ) | ( summer , Thu ) | -74.08 | -4.07 | 23.68 | 124.49 | 7.72 | <0.001 |
| ( spring , Thu ) | ( fall , Thu ) | -15.92 | -4.78 | -34.4 | 66.25 | 1.66 | 0.9 |
| ( summer , Thu ) | ( fall , Thu ) | 58.16 | -0.72 | 7.81 | 108.51 | 6.07 | 0.01 |
| ( winter , Fri ) | ( spring , Fri ) | -31.7 | -9.43 | -18.74 | 82.13 | 3.3 | 0.82 |
| ( winter , Fri ) | ( summer , Fri ) | -60.8 | -3.02 | 10.25 | 111.34 | 6.32 | <0.001 |
| ( winter , Fri ) | ( fall , Fri ) | -27.54 | -12.01 | -22.97 | 78.06 | 2.86 | 0.9 |
| ( spring , Fri ) | ( summer , Fri ) | -29.1 | 6.41 | -21.22 | 79.42 | 3.04 | 0.9 |
| ( spring , Fri ) | ( fall , Fri ) | 4.15 | -2.58 | -46.14 | 54.45 | 0.43 | 0.9 |
| ( summer , Fri ) | ( fall , Fri ) | 33.25 | -8.99 | -17.15 | 83.66 | 3.47 | 0.74 |
| ( winter , Sat ) | ( spring , Sat ) | -41.69 | -10.75 | -8.86 | 92.23 | 4.33 | 0.31 |
| ( winter , Sat ) | ( summer , Sat ) | -81.37 | -7.01 | 30.78 | 131.97 | 8.45 | <0.001 |
| ( winter , Sat ) | ( fall , Sat ) | -17.79 | -18.1 | -32.78 | 68.36 | 1.85 | 0.9 |
| ( spring , Sat ) | ( summer , Sat ) | -39.69 | 3.73 | -10.8 | 90.17 | 4.13 | 0.42 |
| ( spring , Sat ) | ( fall , Sat ) | 23.9 | -7.36 | -26.56 | 74.36 | 2.49 | 0.9 |
| ( summer , Sat ) | ( fall , Sat ) | 63.58 | -11.09 | 13.07 | 114.1 | 6.61 | <0.001 |
| ( winter , Sun ) | ( spring , Sun ) | -28.3 | -11.63 | -22.16 | 78.76 | 2.95 | 0.9 |
| ( winter , Sun ) | ( summer , Sun ) | -69.5 | -5.42 | 18.98 | 120.02 | 7.23 | <0.001 |
| ( winter , Sun ) | ( fall , Sun ) | -18.15 | -6.1 | -32.28 | 68.58 | 1.89 | 0.9 |
| ( spring , Sun ) | ( summer , Sun ) | -41.2 | 6.2 | -9.18 | 91.58 | 4.3 | 0.33 |
| ( spring , Sun ) | ( fall , Sun ) | 10.15 | 5.52 | -40.14 | 60.45 | 1.06 | 0.9 |
| ( summer , Sun ) | ( fall , Sun ) | 51.35 | -0.68 | 1 | 101.7 | 5.36 | 0.04 |

** p<0.001 significant
